# Cholesterol Efflux Capacity of Apolipoprotein A-I Varies with the Extent of Differentiation and Foam Cell Formation of THP-1 Cells

**DOI:** 10.1155/2016/9891316

**Published:** 2016-11-09

**Authors:** Kouji Yano, Ryunosuke Ohkawa, Megumi Sato, Akira Yoshimoto, Naoya Ichimura, Takahiro Kameda, Tetsuo Kubota, Minoru Tozuka

**Affiliations:** ^1^Analytical Laboratory Chemistry, Field of Applied Laboratory Science, Graduate School of Health Care Sciences, Tokyo Medical and Dental University, 1-5-45 Yushima, Bunkyo-ku, Tokyo 113-8519, Japan; ^2^Department of Medical Technology, School of Health Sciences, Tokyo University of Technology, 5-23-22 Nishi-Kamata, Ohta-ku, Tokyo 144-8535, Japan; ^3^Microbiology and Immunology, Field of Applied Laboratory Science, Graduate School of Health Care Sciences, Tokyo Medical and Dental University, 1-5-45 Yushima, Bunkyo-ku, Tokyo 113-8519, Japan

## Abstract

Apolipoprotein A-I (apoA-I), the main protein component of high-density lipoprotein (HDL), has many protective functions against atherosclerosis, one of them being cholesterol efflux capacity. Although cholesterol efflux capacity measurement is suggested to be a key biomarker for evaluating the risk of development of atherosclerosis, the assay has not been optimized till date. This study aims at investigating the effect of different states of cells on the cholesterol efflux capacity. We also studied the effect of apoA-I modification by homocysteine, a risk factor for atherosclerosis, on cholesterol efflux capacity in different states of cells. The cholesterol efflux capacity of apoA-I was greatly influenced by the extent of differentiation of THP-1 cells and attenuated by excessive foam cell formation.* N*-Homocysteinylated apoA-I indicated a lower cholesterol efflux capacity than normal apoA-I in the optimized condition, whereas no significant difference was observed in the cholesterol efflux capacity between apoA-I in the excessive cell differentiation or foam cell formation states. These results suggest that cholesterol efflux capacity of apoA-I varies depending on the state of cells. Therefore, the cholesterol efflux assay should be performed using protocols optimized according to the objective of the experiment.

## 1. Introduction

Atherosclerosis is a major trigger for cardiovascular and cerebrovascular diseases. Since these are the leading causes of death in the developed countries [1], a prevention of atherosclerosis is very crucial. In humans, many defense mechanisms exist against the development of atherosclerosis, and apolipoprotein A-I (apoA-I), the major protein component of high-density lipoprotein (HDL), is associated with some of these mechanisms. Antioxidant [2, 3] and anti-inflammatory abilities [2, 4, 5] and cholesterol efflux capacity [6, 7] are widely recognized as antiatherosclerotic functions of apoA-I. Cholesterol efflux plays a particularly important role in the prevention of progression of atherosclerotic lesions since excessive lipids, such as cholesterol, are removed from the atherosclerotic lesions by apoA-I via the ATP-binding cassette transporter A1 (ABCA1). Recently, studies have reported that the cholesterol efflux capacity is inversely associated with the occurrence of cardiovascular events [8–10].

It is known that apoA-I, which plays a central role in cholesterol efflux capacity, is modified by various reactions such as oxidation, glycation, and homocysteinylation [11–15]. These modifications of apoA-I, presumed to lead to the development of atherosclerosis, have been the focal point of many studies investigating dysfunctionalities in cholesterol efflux. Actually, there are various studies on the relationship between modification of apoA-I and cholesterol efflux capacity that have been reported [16–18]. However, the assay for measurement of cholesterol efflux capacity has not been optimized even by using the same cell line and it is not known whether cellular conditions could lead to a difference in cholesterol efflux capacity. Therefore, in this study, we assessed the cholesterol efflux capacity of apoA-I by using various states of cells.

Moreover, we focused on the role of homocysteine (Hcy), one of the risk factors for atherosclerosis, in cholesterol efflux [19, 20]. Hcy is a metabolite of methionine, an essential amino acid, and is converted to methionine and cysteine [21]. A part of Hcy is converted to homocysteine thiolactone (HcyT) by methionyl-tRNA synthetase [22]. HcyT is rich in reactivity and is known to bind to lysine residues (*N*-homocysteinylation) of many proteins in normal human plasma such as albumin, hemoglobin, and fibrinogen [23]. We recently reported that* N*-homocysteinylated apoA-I (*N*-Hcy apoA-I) exists in normal human serum in the ratio of 1.0–7.4% to total apoA-I [24]. However, the effect of* N*-homocysteinylation of apoA-I on the cholesterol efflux capacity is not yet confirmed. We also evaluated the cholesterol efflux capacity of* N*-Hcy apoA-I using various states of cells and compared the results to that obtained by using normal apoA-I.

## 2. Materials and Methods

### 2.1. Chemicals and Blood Samples

Unless otherwise stated, all reagents were purchased from Wako Pure Chemical. Blood samples were obtained from healthy volunteers after their informed consent, and the study was approved by the ethics committee of Tokyo Medical and Dental University (number 1441).

### 2.2. Cell Culture

THP-1, a human monocyte cell line, was purchased from ATCC and maintained in RPMI-1640 (Sigma-Aldrich) containing 10% fetal bovine serum (FBS), 0.1% penicillin/streptomycin, and 0.1% nonessential amino acids at 37°C under 5% CO_2_.

### 2.3. Cholesterol Efflux Capacity

**Figure 1:**
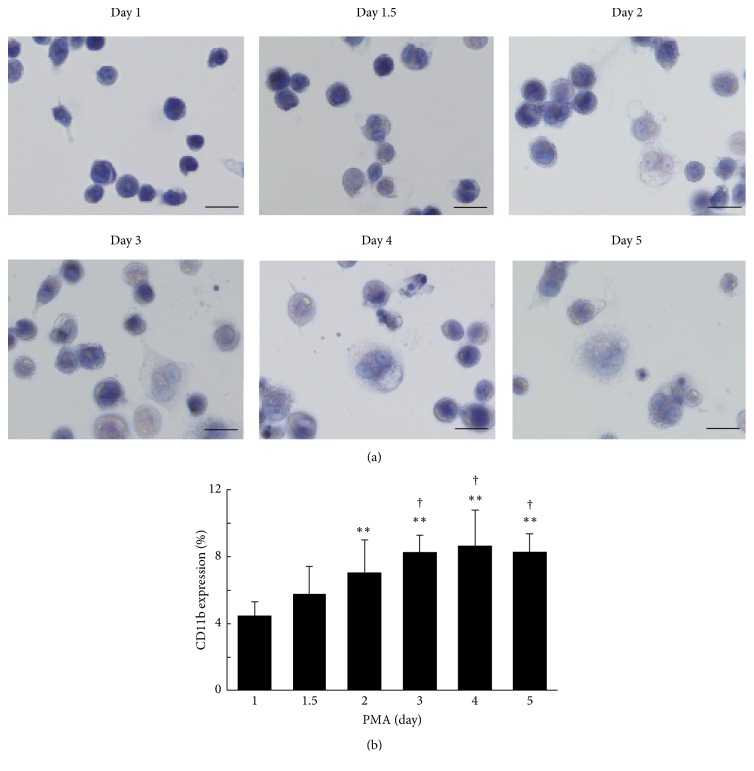
The morphological changes in THP-1 cells. THP-1 cells were treated with 100 ng/mL PMA for different duration (1 to 5 days). After treatment, (a) THP-1 cells were fixed with 3.7% formaldehyde and stained with hematoxylin (scale bars, 20 *μ*m). (b) THP-1 cells were incubated with FITC-conjugated anti-CD11b antibody, and CD11b expression was analyzed using flow cytometry. The percentage of CD11b positive cells which exceeded the maximum fluorescent intensity of the isotype control in each day was indicated. The value at day 1 was defined as 1. The values were indicated by mean + SD (*n* = 5, ^*∗∗*^ *P* < 0.01 versus day 1, ^†^ *P* < 0.05 versus day 1.5).

THP-1 cells were incubated with RPMI-1640 medium containing 100 ng/mL of phorbol 12-myristate 13-acetate (PMA; Sigma-Aldrich) supplemented with 0.2% bovine serum albumin (BSA) in 24-well cell culture plates at a density of 2.5 × 10^5^ cells/well. For preparation of various states of differentiated cells, the incubation time was varied from 1 to 5 days as indicated in Figure 1(a). After removing the supernatant, THP-1 cells were loaded with RPMI-1640 containing acetylated low-density lipoprotein (acLDL) (50 *μ*g protein/mL), T0901317 (1 mmol/L; Enzo Life Sciences), a Liver X receptor (LXR) agonist for promoting expression of ABCA1, ^3^H-cholesterol (1 *μ*Ci/mL; PerkinElmer), and BSA (0.2%). The loading time was also varied over 1 to 5 days to generate different stages of foam cells. THP-1 cells were washed thrice with RPMI-1640 medium containing 0.2% BSA before equilibration with RPMI-1640 supplemented with T0901317 (1 mmol/L) and BSA (0.2%) for 18 hours. After removing the supernatant, cells were incubated with apoA-I (10 *μ*g/mL) and T0901317 (1 mmol/L) in RPMI-1640 for additional 4 hours. Efflux medium was separated from the cells and NaOH (0.1 mol/L) was added directly to the cells for lysis. Both the media and cell lysates were counted for radioactivity using the liquid scintillation counter. Cholesterol efflux capacity was calculated as a percentage of radioactivity in the medium as per the given formula: {^3^H-cholesterol in medium/(^3^H-cholesterol in medium + ^3^H-cholesterol in cells)}  × 100 − percentage of passive diffusion in the case of no acceptor (apoA-I).

### 2.4. Morphological Analysis

For microscopic observation, THP-1 cells were differentiated and loaded at a density of 2.5 × 10^5^ cells/well for different time periods on cover glass (Thermo Fisher Scientific) in 24-well cell culture plates. After incubation, adherent cells were washed with phosphate buffered saline (PBS) and then fixed with 3.7% formaldehyde solution. After washing with PBS, differentiated cells were stained with hematoxylin solution, and loaded cells after differentiation were stained with hematoxylin solution followed by working solution of oil red O for assessing the extent of foam cell formation. The plates were washed with distilled water and observed under the microscope (BX53-34-FL, Olympus). A semiquantification of lipid droplets stained by oil red O was performed with ImageJ (National Institutes of Health).

### 2.5. Flow Cytometric Analysis

THP-1 cells were treated with PMA in 6-well culture plates (1.0 × 10^6^ cells/well) for various time periods as described above, and adherent cells were then washed with PBS. After washing, cells were detached gently with a cell scraper and stained with fluorescein isothiocyanate- (FITC-) conjugated anti-CD11b mouse IgG (Abcam, ab25727, monoclonal) (dilution 1 : 100) for 30 min in a dark place at 4°C. After washing, cells were fixed with 3% paraformaldehyde/PBS. To remove aggregates, cells were filtered using a nylon filter and the concentration was adjusted to approximately 1.0 × 10^5^ cells/mL. CD11b expression was analyzed by flow cytometry (Epics XL, Beckman Coulter) with excitation at 505 nm and emission at 545 nm. Samples were controlled by using FITC-conjugated mouse IgG as an isotype control (Abcam, ab91365, monoclonal) in each day and the percentage of cells which exceeded the maximum fluorescent intensity of isotype control was defined as certain CD11b positive cells.

### 2.6. Preparation of LDL, HDL, and apoA-I

LDL (1.019–1.063 g/mL) and HDL (1.063–1.21 g/mL) were isolated from serum obtained from healthy subjects by ultracentrifugation, as described previously [25]. Next, LDL and HDL fractions were dialyzed against PBS. To purify apoA-I, HDL fraction was delipidated with ethanol/ether 3 : 2 (v/v) and applied to S-200-HR (Sigma-Aldrich) column (2.5 × 90 cm), equilibrated with 20 mmol/L Tris-HCl buffer (pH 7.4) containing 6.8 mol/L urea. The isolated apoA-I fraction was dialyzed against PBS and stored at −20°C until use (Supplemental Figure  1 in Supplementary Material available online at http://dx.doi.org/10.1155/2016/9891316). LDL fraction was acetylated as reported previously [26].

### 2.7. Western Blotting

The expression of CD11b after PMA treatment for different time periods was evaluated by western blotting as described previously [24]. THP-1 cells were lysed with radioimmunoprecipitation buffer (RIPA buffer; 50 mmol/L Tris-HCl pH 8.0 including 150 mmol/L sodium chloride, 0.5% sodium deoxycholate, and 1.0% nonidet P-40 (MP Biomedicals)) containing protease inhibitor cocktails (Roche) at 4°C. The amount of total protein in cell lysates was measured by Folin-Lowry method. Cell lysates were separated by sodium dodecyl sulfate-polyacrylamide gel electrophoresis (SDS-PAGE) and transferred onto PVDF membranes (Millipore), which were then incubated with 5% skim milk in Tris-buffered saline containing 0.05% Tween 20 (washing buffer). PVDF membranes were incubated with primary antibodies, anti-CD11b (Abcam, ab52478, monoclonal) and anti-*β*-actin (Santa Cruz, sc-13-656, polyclonal). After three washes with washing buffer, the membranes were incubated with peroxidase-labeled secondary antibody (Beckman Coulter, IM0831, polyclonal). The bands of CD11b and *β*-actin were visualized by ECL Prime western blotting detection reagent (GE Healthcare). Semiquantification was performed by densitometry (CS Analyzer, ATTO).

### 2.8. *N*-Homocysteinylation of apoA-I

*N*-Homocysteinylation of apoA-I was performed according to the previously reported method with a slight modification [15]. The purified apoA-I (70 *μ*mol/L) was incubated with or without 10 mmol/L of HcyT (MP Biomedicals) at 37°C for 6 hours. Then, samples were dialyzed against PBS. To confirm the production of* N*-Hcy apoA-I, samples treated with cysteamine were isolated by isoelectric focusing and transferred onto PVDF membranes, which were then incubated with primary antibody, anti-apoA-I (Academy Biomedical, 11A-G2b, polyclonal). After washing thrice, the membranes were incubated with peroxidase-labeled secondary antibodies (Medical and Biological Laboratories). The bands containing apoA-I were visualized with 3,3′-diaminobenzidine-4HCl and H_2_O_2_, and semiquantified by CS Analyzer.

### 2.9. Statistical Analysis

The differences of cholesterol efflux capacities among states of cells were analyzed using Kruskal-Wallis test followed by a Mann–Whitney *U* test with Bonferroni correction. Comparison of cholesterol efflux capacity between apoA-I and* N-Hcy apoA-I* was evaluated by a Mann–Whitney *U* test. The results are expressed as mean + standard deviation (SD). A *P* value < 0.05 was considered as statistically significant.

## 3. Results

### 3.1. Effect of Differentiation on Cholesterol Efflux Capacity of apoA-I

**Figure 2:**
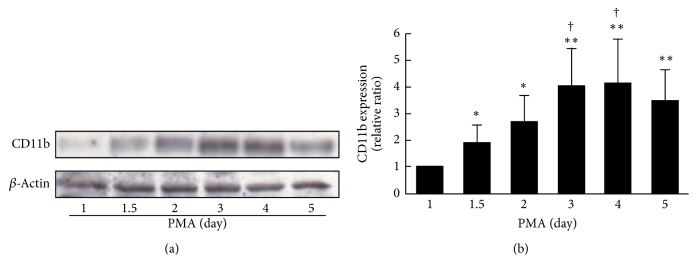
CD11b expression on THP-1 cells treated with PMA for different periods. (a) After PMA treatment for 1 to 5 days, cell lysates (10 *μ*g of proteins) were separated by SDS-PAGE (7% polyacrylamide gel) and detected with anti-CD11b or anti-*β*-actin antibodies. (b) The expression of CD11b also shows bar graphs. The value at day 1 was defined as 1. The values were indicated by mean + SD (*n* = 4, ^*∗*^ *P* < 0.05 versus day 1, ^*∗∗*^ *P* < 0.01 versus day 1, and ^†^ *P* < 0.05 versus day 1.5).

**Figure 3:**
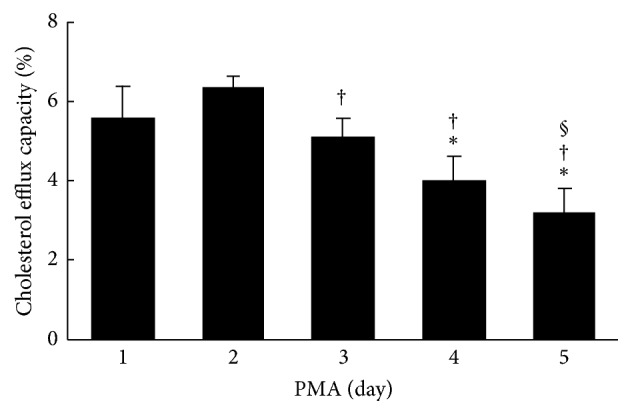
The effect of differentiation on cholesterol efflux capacity of apoA-I. THP-1 cells were treated with 100 ng/mL PMA for different periods (1 to 5 days) and loaded with ^3^H-cholesterol (1 *μ*Ci/mL), acLDL (50 *μ*g protein/mL), and T0901317 (1 *μ*mol/L) for 1 day. The cholesterol efflux capacity of apoA-I (10 *μ*g/mL) was determined under these conditions. The values were indicated by mean + SD (*n* = 3, ^*∗*^ *P* < 0.05 versus day 1, ^†^ *P* < 0.05 versus day 2, and ^§^ *P* < 0.05 versus day 3).

THP-1 cells differentiated following treatment with PMA for 1 to 5 days. To confirm differentiation of the cells at each stage, the morphology of THP-1 cells was assessed by staining with hematoxylin (Figure 1(a)). Mild morphological changes were observed at day 1.5, and most of these were adendritic cells with approximately 20 *μ*m size. From day 1.5 to day 2, dynamic changes in cells were observed. Cells grew larger and showed the appearance of dendrite formation corresponding to the increase in stimulation. Next, we evaluated the expression of CD11b, an adhesion molecule, on THP-1 cells by flow cytometric analysis (Figure 1(b)). The expression of CD11b was essentially increased in a stimulation with PMA on THP-1 macrophages, while the expression on THP-1 monocytes without PMA treatment was not almost detected (data not shown). Conclusively, expression of CD11b at day 2 was significantly higher than that at day 1 (*P* < 0.01) and maintained a plateau state (Figure 1(b)). A similar tendency was also observed in the western blotting analysis (Figures 2(a) and 2(b)). The effect of differentiation of THP-1 cells on cholesterol efflux capacity of apoA-I was evaluated on the condition that the period of foam cell formation was fixed at 1 day. The cholesterol efflux capacities were the highest and the second highest at day 2 and day 1 of differentiation, respectively, and gradually deceased after day 3 (Figure 3). Although the period of foam cell formation was fixed for 1 day, total ^3^H-cholesterol taken up by the cells before efflux was largely different according to the differentiation periods, the lowest level at day 1, a peak at day 2, and gradual decrease after day 3 (Supplementary Figure  2(a)). In addition, ^3^H-cholesterol mass in the medium after efflux also showed a similar tendency to total ^3^H-cholesterol taken up by the cells (Supplementary Figure  2(b)). Cholesterol efflux capacities expressed in percentage were largely different from the actual efflux of ^3^H-cholesterol mass. In contrast, ABCA1 expression was the highest at day 1 and gradually reduced in parallel with the duration of PMA treatment (Supplementary Figure  3).

### 3.2. Effect of Foam Cell Formation on Cholesterol Efflux Capacity of apoA-I

**Figure 4:**
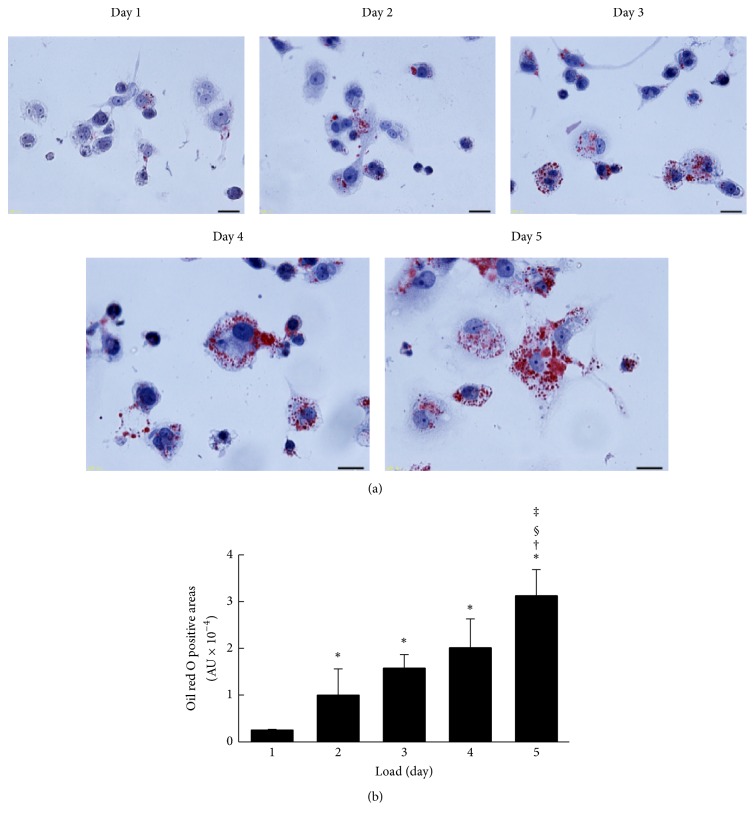
Foam cell formation. (a) After PMA treatment for 2 days, cells were loaded with acLDL (50 *μ*g protein/mL) and T0901317 (1 *μ*mol/L) for indicated periods. After equilibration, cells were stained with oil red O and hematoxylin (scale bars, 20 *μ*m). (b) Semiquantitative analysis of oil red O positive cells was performed using the ImageJ (National Institutes of Health). The values were indicated by mean + SD (*n* = 3, ^*∗*^ *P* < 0.05 versus day 1, ^†^ *P* < 0.05 versus day 2, ^§^ *P* < 0.05 versus day 3, and ^‡^ *P* < 0.05 versus day 4).

**Figure 5:**
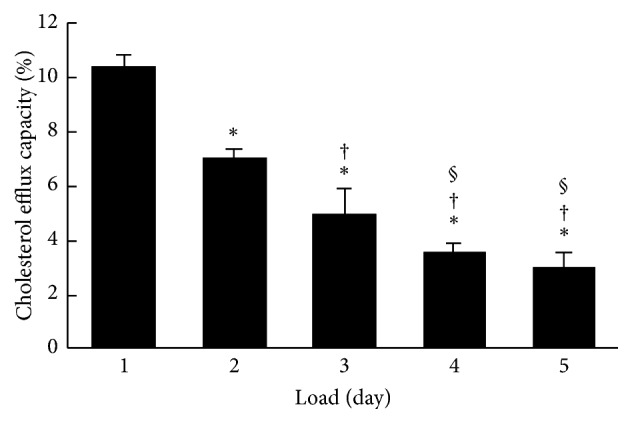
The effect of foam cell formation on cholesterol efflux capacity of apoA-I. After PMA treatment for 2 days, cells were loaded with ^3^H-cholesterol (1 *μ*Ci/mL), acLDL (50 *μ*g protein/mL), and T0901317 (1 *μ*mol/L) for 1 to 5 days. Under these conditions, the cholesterol efflux capacity was determined. The values were indicated by mean + SD (*n* = 3, ^*∗*^ *P* < 0.05 versus day 1, ^†^ *P* < 0.05 versus day 2, ^§^ *P* < 0.05 versus day 3, and ^§^ *P* < 0.05 versus day 3).

In experiments wherein the stage of differentiation of THP-1 cells with PMA treatment was fixed for 2 days, THP-1 macrophages were incubated with acLDL for 1 to 5 days. The accumulations of intracellular lipid droplets were evaluated using oil red O staining (Figure 4(a)). Lipid droplets stained with oil red O were faintly observed from day 1 and dramatically increased depending on the loading period of acLDL (Figure 4(b)). Subsequently, the cholesterol efflux capacities of apoA-I in each stage of cells were determined. Cholesterol efflux capacity was highest for 1 day after loading with acLDL and significantly decreased in a loading time-dependent manner (Figure 5). Cellular uptake of total ^3^H-cholesterol before efflux showed a gradual increase, whereas medium ^3^H-cholesterol after efflux was slightly decreased during acLDL loading (Supplementary Figures  2(c) and 2(d)). In addition, no significant change in ABCA1 expression was observed (Supplementary Figure  4).

### 3.3. Cholesterol Efflux Capacity of* N*-Hcy apoA-I

**Figure 6:**
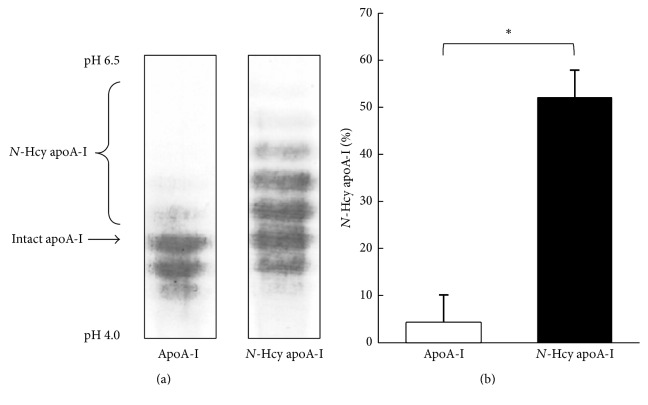
Isoelectric focusing for apoA-I and apoA-I treated with homocysteine thiolactone. (a) Representative isoelectric focusing patterns of apoA-I and* N*-Hcy apoA-I. Purified apoA-I was incubated with or without 10 mmol/L of homocysteine thiolactone at 37°C for 6 hours. After dialysis against PBS, the samples were analyzed by isoelectric focusing followed by western blotting using anti-apoA-I antibody. (b) Histogram represents the percentage of* N*-Hcy apoA-I to total apoA-I using CS analyzer. The values were indicated by mean + SD (*n* = 3, ^*∗*^ *P* < 0.05).

**Figure 7:**
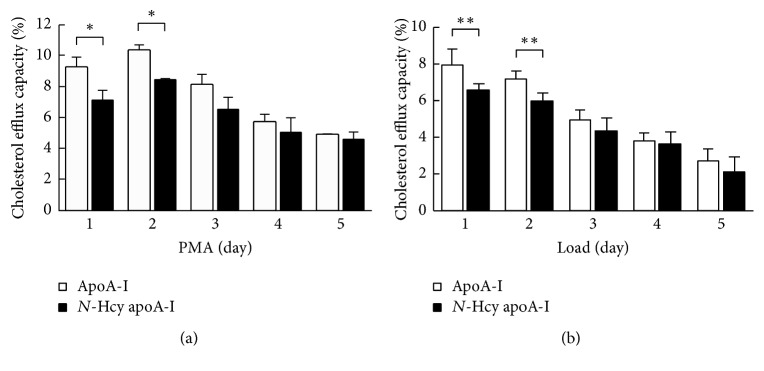
The effect of differentiation and foam cell formation on cholesterol efflux capacity of apoA-I and* N*-Hcy apoA-I. (a) After THP-1 cells were differentiated with PMA for different periods (1 to 5 days) and were loaded with ^3^H-cholesterol (1 *μ*Ci/mL), acLDL (50 *μ*g/mL), and T0901317 (1 *μ*mol/L) for 1 day, cholesterol efflux capacity was assessed by using apoA-I (10 *μ*g/mL) or* N*-Hcy apoA-I (10 *μ*g/mL). The values were indicated by mean + SD (*n* = 3, ^*∗*^ *P* < 0.05). (b) After PMA treatment for 2 days and loading with ^3^H-cholesterol (1 *μ*Ci/mL), acLDL (50 *μ*g/mL), and T0901317 (1 *μ*mol/L) for different periods (1 to 5 days), cholesterol efflux capacity was determined by using apoA-I (10 *μ*g/mL) or* N*-Hcy apoA-I (10 *μ*g/mL). The values were indicated by mean + SD (*n* = 6, ^*∗∗*^ *P* < 0.01).

Using various stages of cells regulated differentiation and foam cell formation, the effect of* N*-homocysteinylation on cholesterol efflux capacity of apoA-I was evaluated. To confirm the extent of* N*-homocysteinylation of apoA-I, apoA-I incubated with HcyT was tested using an isoelectric focusing. Compared to normal apoA-I,* N*-Hcy apoA-I indicates higher isoelectric point (pI) depending on the number of attached homocysteine compounds (Figure 6(a)). The ratio of* N*-Hcy apoA-I to total apoA-I on the isoelectric focusing pattern was 51.9 ± 5.9% (Figure 6(b)). Cholesterol efflux capacities of apoA-I and* N*-Hcy apoA-I reached those peaks at day 2 and gradually decreased depending on the periods of the treatment with PMA (Figure 7(a)). In case of foam cell formation, a significant decrease was observed in cholesterol efflux capacities of apoA-I and* N*-Hcy apoA-I according to the acLDL loading (Figure 7(b)). In addition, the cholesterol efflux capacity of* N*-Hcy apoA-I was significantly lower than that of intact apoA-I by differentiation and foam cell formation at day 1 and day 2, respectively; however no significant difference was observed after day 3 (Figure 7).

## 4. Discussion

Cholesterol efflux is essential for the antiatherosclerotic function of apoA-I, and the evaluation of its capacity would provide important information in terms of prevention and diagnosis of atherosclerosis. However, in previous reports assays for measurement of cholesterol efflux capacity of apoA-I and modified apoA-I have been performed using different methods. Therefore, the effect of modification of apoA-I on cholesterol efflux capacity is often controversial [16–18]. Our aim was to evaluate the variation in cholesterol efflux capacity of apoA-I under various cellular conditions such as differentiation and foam cell formation and to investigate the effect of* N*-homocysteinylation of apoA-I on cholesterol efflux capacity in these conditions. First, we confirmed the morphological features and expression of CD11b, which is an established differentiation marker of macrophages of THP-1 cells treated with PMA [27]. THP-1 cells treated with PMA for 2 days showed a behavior similar to macrophages morphologically, including increase in cell volume [28]. It is known that PMA induces CD11b expression via protein kinase C delta (PKC-*δ*) [27]. An increase in the expression of CD11b on THP-1 cells treated with PMA for 2 days was observed by flow cytometric and western blotting analysis while the expression of ABCA1 decreased from day 1 to day 5 (Supplementary Figure  3). PMA is presumed to have reduced the ABCA1 gene expression via PKC activation [29], since PKC was activated with PMA as stated above. Taken together, these results suggest that variously differentiated cells were prepared. Second, we evaluated the effect of differentiation of THP-1 cells on cholesterol efflux capacity. Cholesterol efflux capacity reached a peak at day 2 and decreased after day 3 (Figure 3). At day 1, the uptake of ^3^H-cholesterol into cells and levels detected in the medium were low (Supplementary Figures  2(a) and 2(b)). At day 2, the uptake of intracellular ^3^H-cholesterol was about three times as high as at day 1. On the contrary, after day 3, both intracellular and medium ^3^H-cholesterol were decreased, and the decreased ratios of medium ^3^H-cholesterol were slightly larger than those of intracellular ^3^H-cholesterol. These results suggest that the increase of the cholesterol efflux capacity indicated as a percentage at day 2 was caused by the differentiation of THP-1 macrophages, and a decrease after day 3 would be determined by the reduced amount of the expression of ABCA1, a pivotal transporter in cholesterol efflux to apoA-I. In many studies, the duration of PMA stimulation has been inconsistent for the evaluation of cholesterol efflux capacity [16–18]. Our results indicate that the optimum period of PMA treatment is 2 days if the cholesterol efflux capacity is required to be assessed at the maximum stage.

Next, we examined the effect of foam cell formation on cholesterol efflux capacity. Oil red O positive cells, which indicate the formation of lipid droplets, were increased in proportion to days of loading acLDL (Figure 4). Meanwhile, cholesterol efflux capacity was decreased in a loading time-dependent manner (Figure 5). Although there was an increase in the intracellular ^3^H-cholesterol over the passage of days, the levels of secreted ^3^H-cholesterol in the medium by apoA-I were decreased gradually (Supplementary Figures  2(c) and 2(d)). Moreover, no significant change was observed in ABCA1 expression (Supplementary Figure  4). The excessive accumulation of cholesterol ester in cells is reported to inhibit lysosomal acid lipase (LAL) activity, an enzyme related to the cholesterol metabolism [30]. Free cholesterol taken up into cells is esterified by acyl coenzyme-A cholesterol acyltransferase (ACAT) and stored as lipid droplets [31]. Since cholesterol ester is hydrolyzed by LAL and removed as free cholesterol through ABCA1, the inhibition of LAL may lead to a reduction in the cholesterol efflux regardless of the degree of ABCA1 expression on the cells surface. Hence, decrease in cholesterol efflux without reduction of ABCA1 expression may be related to the loss of LAL activity caused by the excessive uptake of acLDL, including ^3^H-cholesterol, and the effect might be reversed in the presence of an ACAT inhibitor. These results indicate that the optimum period of loading cholesterol is 1 day if the cholesterol efflux capacity has to be assessed at the maximum stage. Longer durations of loading cholesterol decrease cholesterol efflux capacity because of excessive cholesterol accumulation.

To the best of our knowledge, this study is the first to indicate the relationship between cholesterol efflux capacity and condition of THP-1 cells such as differentiation and foam cell formation, in depth. Using these different states of cells, we examined the effect of* N*-homocysteinylation on cholesterol efflux capacity of apoA-I.* N*-Hcy apoA-I was detected as a more positively charged band than intact apoA-I (Figure 6) and was defined as a percentage to total apoA-I, as described previously [15]. The variation in cholesterol efflux capacities of both apoA-I and* N*-Hcy apoA-I using THP-1 cells treated with PMA in different time intervals indicates similar trends, as compared to the values that reached the peaks at day 2 and then gradually decreased (Figure 7(a)). However, significant differences were observed in the cholesterol efflux capacities between apoA-I and* N*-Hcy apoA-I at day 1 and day 2. On the contrary, no significant difference was observed in those capacities at day 3 and after. Furthermore, cholesterol efflux capacities of both apoA-I and* N*-Hcy apoA-I were also evaluated using THP-1 cells treated with cholesterol loading at different time intervals. Similarly, the cholesterol efflux capacities of these two types of apoA-I showed the same behavior, and* N*-Hcy apoA-I indicated significantly lower capacities than apoA-I at day 1 and day 2 after loading. Only a few studies have been reported about the relationship between Hcy and cholesterol efflux capacity [32, 33]. We previously reported that the cholesterol efflux capacity of apoA-I was not significantly reduced with* N*-homocysteinylation [15]. In the previous study the period of PMA stimulation was 3 days, indicating that the cells might be in the state of excessive differentiation to consider the possible effect of* N*-homocysteinylation. To confirm the effect of* N*-homocysteinylation on cholesterol efflux capacity of apoA-I, the influence of modification site of apoA-I and its dose response should be investigated. However, our results in this study reiterate the importance of the cell conditions such as the degree of differentiation and foam cell formation when the effects of any modifications of apoA-I, not limited to* N*-homocysteinylation, on cholesterol efflux capacity are evaluated. Optimization of experimental conditions will be required depending on the purpose of the measurement. However, we have focused on THP-1 cells in this study. Further investigations are needed to assess the cholesterol efflux capacity using other monocytes or monocytic cell lines [34–40]. Although this study has only examined apoA-I, studies using HDL could be necessary to confirm a precise mechanism. Further experiments will provide us a hint for evaluation of various stages of atherosclerosis.

## 5. Conclusions

In conclusion, the cholesterol efflux capacities of apoA-I and* N*-Hcy apoA-I vary depending on the state of the cells; hence, it is important to evaluate the cholesterol efflux capacity in several states of the cells adapted for a purpose.

## Supplementary Material

Supplementary Fig1: The purity of apoA-I.
Supplementary Fig2: The amount of Total and Medium ^3^H-cholesterol.
Supplementary Fig3: ABCA1 expression on THP-1 cells treated with PMA for different periods.
Supplementary Fig4: ABCA1 expression on THP-1 cells loaded with acLDL for different periods.
