# Supplementary material for: Cholesterol Efflux Capacity of Apolipoprotein A-I Varies with the Extent of Differentiation and Foam Cell Formation of THP-1 Cells
Source: J Lipids. 2016 Nov 9;2016:9891316. doi: 10.1155/2016/9891316 (PMC5120203; doi:10.1155/2016/9891316)
Supplement: Supplementary file 1 — Supplementary Fig1: The purity of apoA-I. Supplementary Fig2: The amount of Total and Medium 3H-cholesterol. Supplementary Fig3: ABCA1 expression on THP-1 cells treated with PMA for different periods. Supplementary Fig4: ABCA1 expression on THP-1 cells loaded with acLDL for different periods. [file 9891316.f1.pdf]

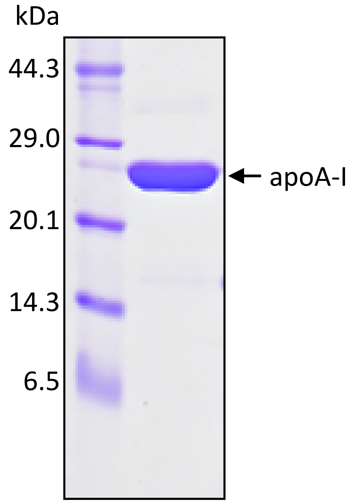

HDL was isolated from serum by ultracentrifugation and delipidated with ethanol/ether. Sample was applied to S-200-HR column and apoA-I fraction was isolated. The fraction was subjected to 12.5% SDS-PAGE (6 μg protein/lane) followed by staining with coomassie brilliant blue (CBB).

Supplementary Figure 2

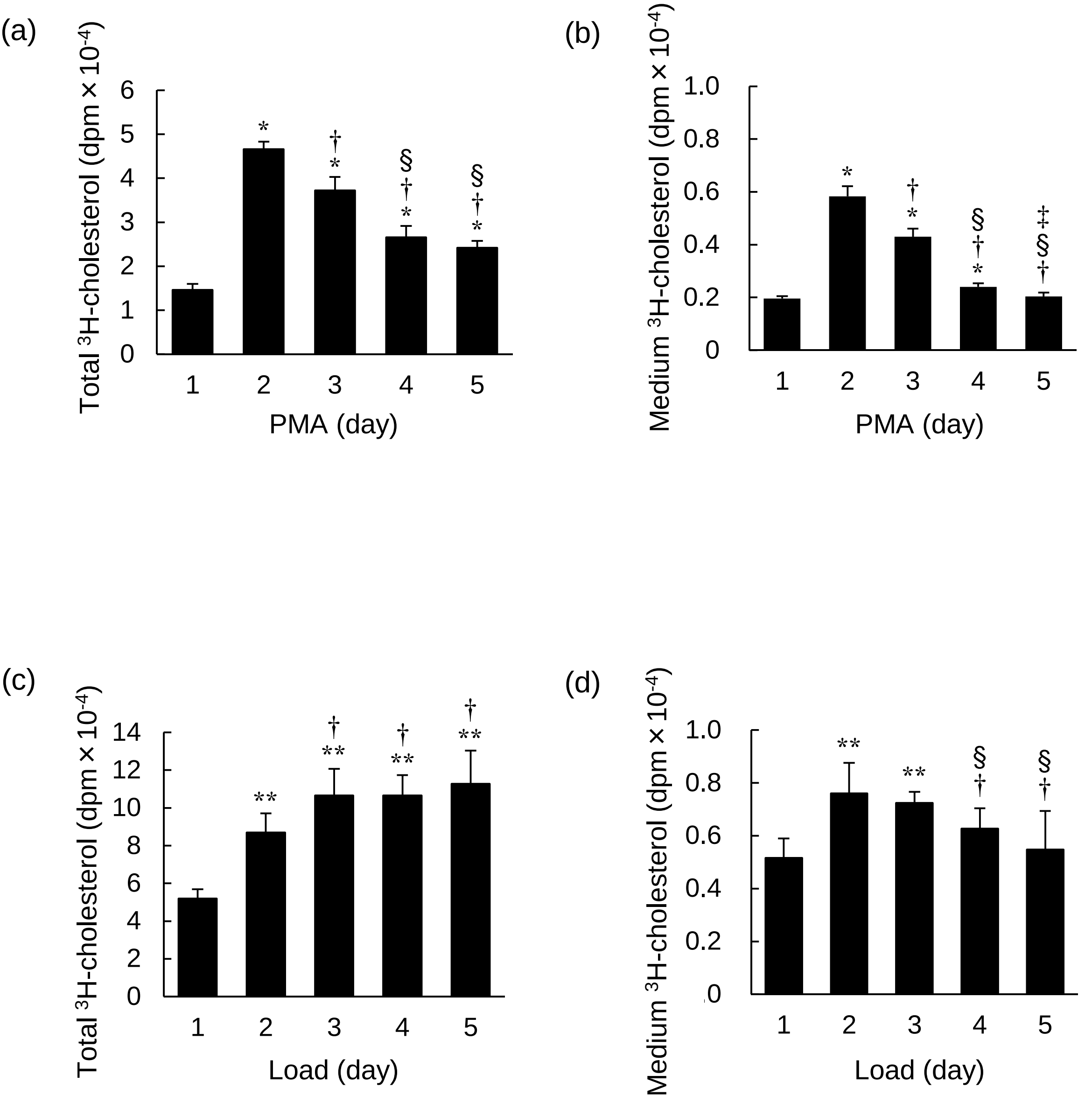

A graph shows the relationship between Total ^{3}H-cholesterol or Medium ^{3}H-cholesterol and period of PMA treatment (a, b) or Load treatment (c, d). The values were indicated by mean＋SD (n=3 (a, b) or n = 6 (c, d), $^{*}P < 0.05$ versus day 1, $^{**}P < 0.01$ versus day 1, $^{\dagger}P < 0.05$ versus day 2, $^{\S}P < 0.05$ versus day 3, $^{\ddagger}P < 0.05$ versus day 4).

Supplementary Figure 3

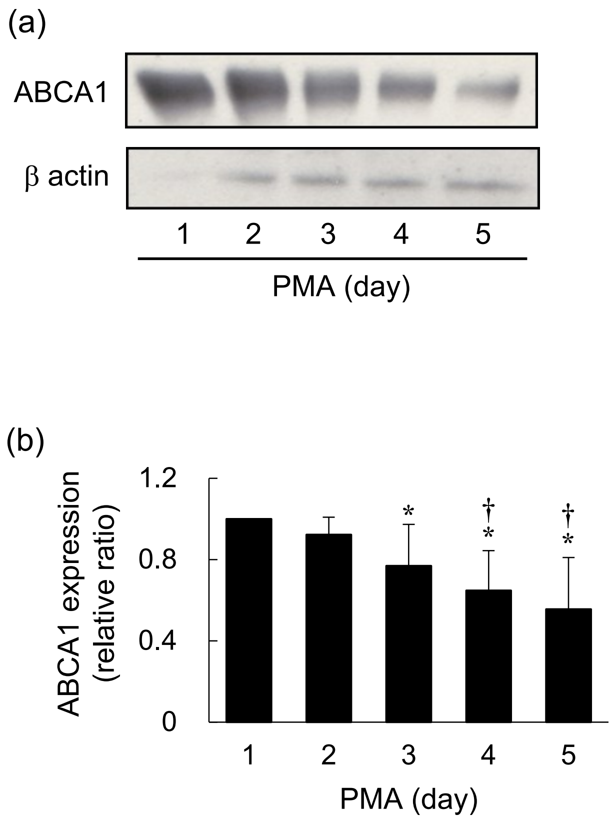

(a) THP-1 cells were treated with 100 ng/mL PMA for 1 to 5 days and loaded with acLDL (50 μg/mL) and T0901317 (1 μmol/L) for 1 day. After equilibration, cells were lysed using RIPA buffer containing protease inhibitors. Then lysates (4 μg/lane) were separated by SDS-PAGE (7% polyacrylamide gel) and detected with ABCA1 or β-actin antibodies. (b) The expression of ABCA1 also shows bar graphs for each day relative to day 1. The values were indicated by mean+SD (n=3 *$P < 0.05$ versus day 1, †$P < 0.05$ versus day 2).

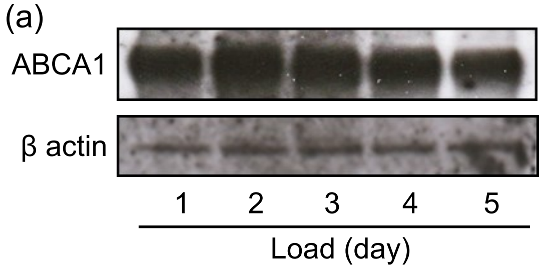

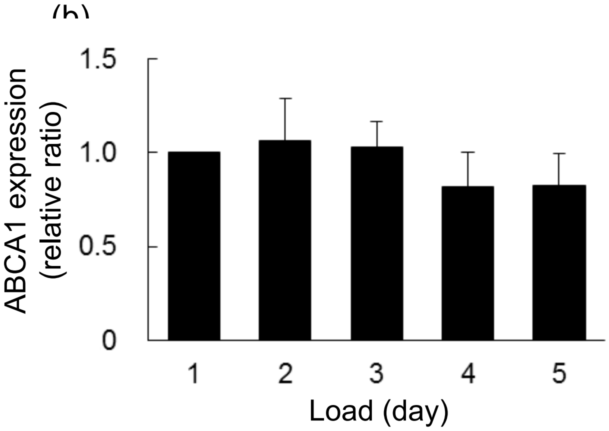

(a) After PMA treatment for 2 days, THP-1 cells were loaded with acLDL (50 μg/mL) and T0901317 (1 μmol/L) for different periods (1 to 5 days). After equilibration, cells were lysed using RIPA buffer containing protease inhibitors. Then lysates (4 μg/lane) were separated by SDS-PAGE (7% polyacrylamide gel) and detected with ABCA1 or β-actin antibodies. (b) The expression of ABCA1 also shows bar graphs for each day relative to day 1. The values were indicated by mean＋SD (n=3).
